# Modified implantation of a Bolton Relay branch arch device into the ascending aorta close to a mechanical aortic valve

**DOI:** 10.1093/icvts/ivac017

**Published:** 2022-02-10

**Authors:** Artur Milnerowicz, Tomasz Jędrzejczak, Paweł Rynio, Arkadiusz Kazimierczak

**Affiliations:** 1 Kliniczny Oddział Chirurgii Naczyniowej 4WSK z Polikliniką Wrocław ul, . Weigla 5, Poland; 2 Klinika Chirurgii Naczyniowej, Ogólnej i Angiologii PUM w Szczecinie. Ul, . Powstańców Wielkopolskich 72, Poland

**Keywords:** Physician Modified Endograft, Externalized Transapical Guidewire, Branch Arch Device, Artificial Aortic Valve

## Abstract

An aortic arch stent graft is usually contraindicated with a mechanical aortic valve. However, a modified stent graft plus the use of an externalized transapical guidewire technique allowed a safe implantation close to a mechanical aortic valve.

## INTRODUCTION

Implanting a Bolton Relay branch device plus a custom-made double-branch endoprosthesis (Bolton Medical/Terumo, Sunrise, FL, USA) into the ascending aorta usually requires the placement of a stiff guidewire and of the delivery system tip into the left ventricle. A mechanical aortic valve impedes this procedure because it blocks the valve discs, causing acute aortic regurgitation, and may fracture the discs [1].

Case report: A 75-year-old man with a residual arch dissection after a type A Bentall open repair was disqualified from open arch surgery [2] because he had a false lumen (FL) that was degenerating rapidly at a rate of >10 mm/year. We obtained written informed consent from the patient and agreement from the hospital bioethical board to proceed with this procedure. To obtain a short distance between the coronary artery bypass and the aortic valve, 15 mm of the distal end of the Relay stent-graft tip (made of a flexible plastic) was cut off (Fig. 2A). This modification must be performed by a surgeon and cannot at present be performed by the company.

A left subclavian artery bypass was performed 4 weeks prior to the stent graft implant. Before beginning the implant procedure, the patient was administered general anaesthesia. Cerebral monitoring was performed (INVOS Cerebral Oximetry System, Medtronic, Inc. Minneapolis, MN, USA). One femoral artery was identified, and 2 Perclose-ProGlide (Abbott Laboratories, Abbott Park, IL, USA) systems were installed followed by a 24 Fr sheath (Sentrant, Medtronic, Dublin, Ireland). A 5 Fr sheath was inserted into the contralateral femoral artery for angiographic monitoring. Both common carotid arteries were exposed. Heparin was given to achieve an activated clotting time ≥300 s.

## MODIFICATION OF AN EXTERNALIZED TRANSAPICAL GUIDEWIRE TECHNIQUE

**Figure 1:**
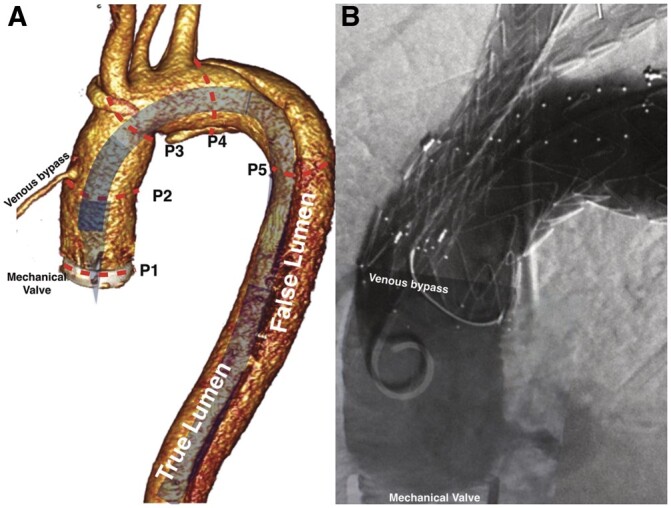
(**A)** 3-Dimensional reconstruction of the tomographic image. Expected position of a delivery system without tip modification. Sizing: P1–P2: space for the tip of the delivery system (29 mm); P2-P3: proximal sealing zone (34 mm); P3-P4: access to the branches (50 mm); P4-P5: distal sealing zone. (**B)** Completion angiographic scan: patent venous bypass; no endoleak.

**Figure 2:**
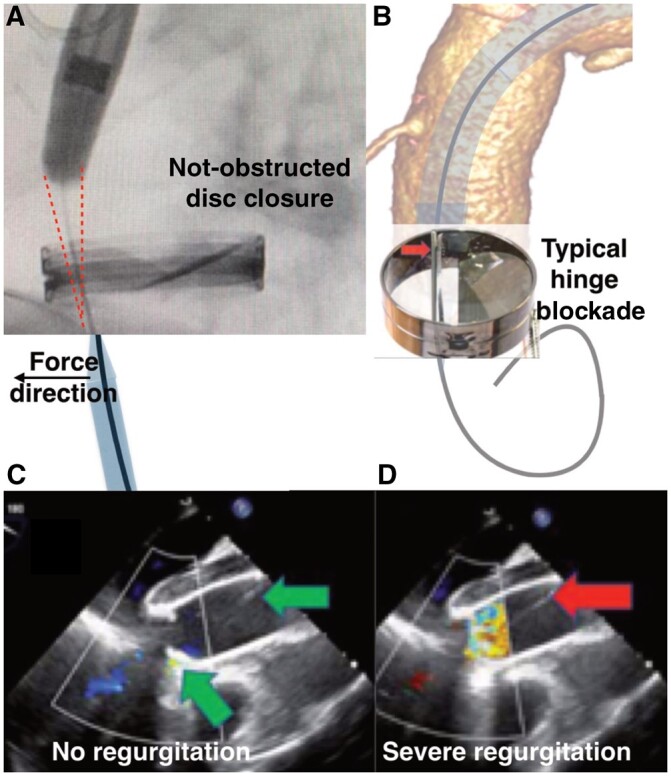
(**A**) Red sketch on the fluoroscopic image shows space obtained after cutting 15 mm from the tip of the stent graft. The wire was guided by the 6 Fr sheath from the apex to keep it away from the disc hinge. (**B)** 3-Dimensional reconstruction of the tomographic image. Mechanism of the disc blockade if the valve is simply crossed. (**C/D)** Transoesophageal echocardiography. (**C)** Effective valve closure; green arrows show correct position of the wire. (**D)** Severe valve regurgitation after the blockade of the disc. Red arrow shows the position of the wire.

Instead of positioning a stiff guidewire in the left ventricle, we used a through-and-through technique over a soft Terumo guidewire. A short incision in the fifth left intercostal space exposed the apex of the heart; a 6 Fr sheath was introduced into the left ventricle. We passed by the valve discs under transoesophageal echocardiography (TOE) monitoring. The position of the soft guidewire was constantly corrected by the 6 Fr sheath and was kept far from the valve hinges and the dics themselves, allowing them to close freely (Fig. 2A). This process did not interfere with their opening while they were being monitored by TOE (Fig. 2C) [3]. The Terumo soft guidewire was snared to the groin. The delivery system passed from the groin into the ascending aorta (the “through-and-through” technique). The guidewire inside the valve was easily steerable at all times. No symptoms of regurgitation or valve dysfunction were noted as the device was implanted. The stent graft was deployed correctly with angiographic monitoring and after rapid pacing. The proximal sealing zone between the venous bypass and the innominate artery had to fit precisely (Fig. 1A). The soft guidewire was then removed from the apex and the valve. The Relay inner branches were extended by bridging limbs. A final angiographic scan confirmed that the device was implanted correctly (Fig. 1B). The apex was closed as usual with the use of 2 slipper stitches on felt pads. Heparin effects were reversed with protamine sulfate 1:1. Pericardial and left pleural drainage was maintained for 2 days. The patient was discharged without neurological deficits on postoperative day 6. A follow-up computed tomography scan after 6 months showed no flow in the false lumen of the aortic arch and the patency of the cephalic vessels. At the subsequent follow-up examinations, the distal dissection had not progressed.

## DISCUSSION

Open surgery is the gold standard treatment for the ascending aorta, but endovascular procedures are increasingly preferred [2] for the 35% of patients considered suitable [4]. Using a stent graft in the ascending aorta is limited by the 40-mm aortic diameter and the length of the proximal landing zone (meaning the distance from the coronary arteries to the innominate artery), usually ≥65 mm [5]. The presence of an aortic mechanical valve plus coronary artery bypass grafts originating from the ascending aorta creates a serious limitation for a stent graft implant in the ascending aorta. According to the manufacturer, the use of a COOK device with a bullet-nose short tip (35 mm) application was not possible, because the length of the proximal landing zone was limited by the CABG and the mechanical valve [6]. Consequently, without the modification to the tip, the inner tunnel outlets would have ended up at the level of the origin of the innominate artery, thereby complicating the BCT branch implant and perfusion of the brain. Moreover, we could not maintain acceptable valve function after performing a simple crossing with the guidewire because the location of the hinge axis was towards the axis of the curve of the arch. Without the modifications described here, a guidewire placed along the main curve would always position itself on the valve hinge (Fig. 2B) and immediately block it, leading to severe valve regurgitation (Fig. 2D). According to Konstantinou *et al.*, it is sometimes possible to pass the artificial aortic valve through its lateral side with the tip of the delivery system and avoid acute left ventricular dilatation [7]. Despite the risk of valve leaflet fracture, it is possible that the presence of the tip inside the valve annulus could have reduced the back flow to the ventricle sufficiently that it would not have triggered acute insufficiency. However, because our patient did not tolerate a half valve blockade, we passed the valve actively, avoiding compression of the leaflet by the guidewire. This step allowed the safe delivery of the stent graft to the aortic arch with the reduced risk of damage to the valve and the assurance that the patient could tolerate it.

## LIMITATION

This technique requires a cardiac surgeon experienced in transapical accesses and TOE. The radio-opaque (carbon) intercostal retractor is recommended.

## CONCLUSION

Modifications to the Bolton Relay arch device and the implant technique allowed a safe implant in a patient with an artificial aortic valve.

## FUNDING

None.

**Conflicts of interest:** None.
